# Bud14 function is crucial for spindle pole body size maintenance

**DOI:** 10.55730/1300-0152.2702

**Published:** 2024-08-05

**Authors:** Sevilay Münire GİRGİN, Ayşe KOCA ÇAYDAŞI

**Affiliations:** Department of Molecular Biology and Genetics, Collage of Sciences, Koç University, İstanbul, Turkiye

**Keywords:** Spindle pole body, Bud14, Glc7, Protein Phosphatase 1, Spc110, centrosome

## Abstract

**Background/aim:**

Spindle pole bodies (SPB), the functional equivalent of centrosomes in yeast, duplicate through generation of a new SPB next to the old one. However, SPBs are dynamic structures that can grow and exchange, and mechanisms that regulate SPB size remain largely unknown. This study aims to elucidate the role of Bud14 in SPB size maintenance in *Saccharomyces cerevisiae*.

**Materials and methods:**

We employed quantitative fluorescence microscopy to assess the relative and absolute amounts of SPB structural proteins at SPBs of wildtype cells and in cells lacking *BUD14* (*bud14Δ*). Quantifications were performed using asynchronous cell cultures, as well as cultures synchronously progressing through the cell cycle and upon different cell cycle arrests. We also utilized mutants that allow the separation of Bud14 functions.

**Results:**

Our results indicate that higher levels of SPB inner, outer, and central plaque proteins are present at the SPBs of *bud14Δ* cells compared to wildtype cells during anaphase, as well as during nocodazole-induced M-phase arrest. However, during α-factor mediated G1 arrest, inner and outer plaque proteins responded differently to the absence of *BUD14*. A Bud14 mutant that cannot interact with the Protein Phosphatase 1 (Glc7) phenocopied *bud14Δ* in terms of SPB-bound levels of the inner plaque protein Spc110, whereas disruption of Bud14-Kel1-Kel2 complex did not alter Spc110 levels at SPBs. In cells synchronously released from α-factor arrest, lack of Bud14-Glc7 caused increase of Spc110 at the SPBs at early stages of the cell cycle.

**Conclusion:**

We identified Bud14 as a critical protein for SPB size maintenance. The interaction of Bud14 with Glc7, but not with the Kelch proteins, is indispensable for restricting levels of Spc110 incorporated into the SPBs.

## 1. Introduction

Most eukaryotic cells utilize microtubule organizing centers called centrosomes to build the mitotic spindle. Like DNA, centrosomes duplicate during the cell cycle, giving rise to two centrosomes that form the two poles of the mitotic spindle. Abnormalities in centrosome number, size, and structure lead to defects in the mitotic spindle and consequently contribute to aneuploidy and chromosome instability, which are hallmarks of cancer (Pihan et al., 2003; Godinho and Pellman, 2014; Schnerch and Nigg, 2016). In addition to microtubule nucleation, centrosomes serve as signaling platforms regulating key cellular pathways, including those that control cell cycle progression, DNA damage response, and development in yeast and higher organisms (Arquint et al., 2014; Gryaznova et al., 2016; Chan et al., 2017; Langlois-Lemay and D’Amours, 2022; Lin et al., 2022).

**Figure 1:**
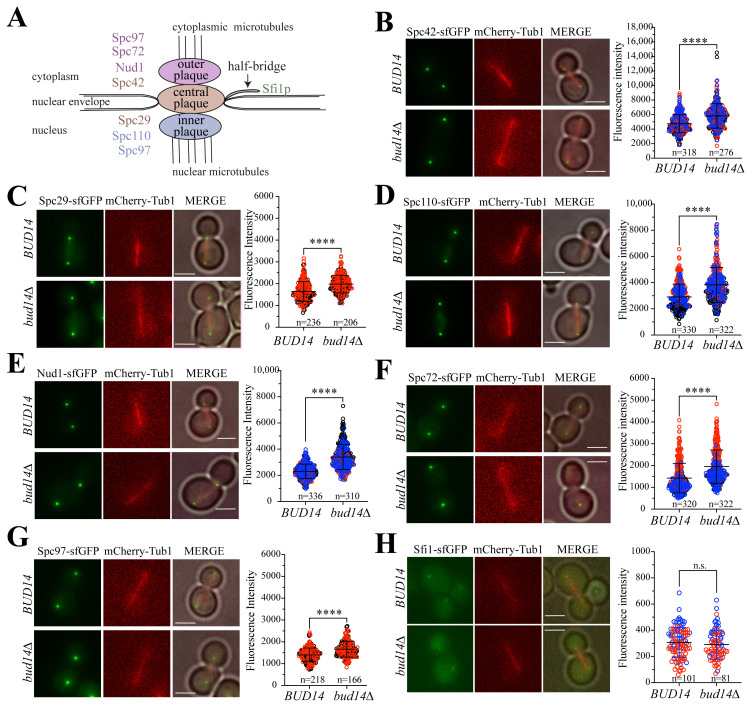
SPB-bound levels of SPB structural proteins in *bud14Δ* cells. **A.** Cartoon depicting SPB structure. Proteins analyzed from the outer, central, and inner plaque, as well as the half-bridge are indicated in colors. Other proteins are omitted for simplicity. **B–H.** Mean fluorescence intensities of SPB-bound Spc42 (B), Spc29 (C), Spc110 (D), Nud1 (E), Spc72 (F), Spc97 (G), Sfi1 (H) in *BUD14* wildtype and in *bud14Δ* cells during anaphase (spindle length ≥ 3μm). Representative images of each strain are shown. mCherry-Tub1 serves as the spindle marker. Scale bar: 3 μm. Red, blue, and black circles shown in the same graph indicate results from independent experiments. Three independent experiments were performed in B, D, E, and F. Two independent experiments were performed in C, G, and H. n: sample size. ****: p < 0.0001 according to two-tailed Student’s *t*-test. n.s.: nonsignificant, p > 0.05.

Spindle pole bodies (SPBs) are the functional equivalent of centrosomes in the budding yeast, *Saccharomyces cerevisiae*. Although SPBs are structurally distant from mammalian centrosomes, homologs of some SPB structural proteins are present at the pericentriolar material region of mammalian centrosomes (Fraschini, 2018; Ito and Bettencourt-Dias, 2018). The SPB is a multilayered structure embedded in the nuclear envelope (Figure 1a), consisting of outer and inner plaques that organize cytoplasmic and nuclear MTs, an inner plaque that connects outer and inner plaques, and the half-bridge that is attached to the central plaque (Cavanaugh and Jaspersen 2017; Viswanath et al., 2017).

SPBs duplicate once and only once per cycle. The first step in SPB duplication is the conversion of the half-bridge to the bridge during late mitosis (Li et al., 2006; Burns et al., 2015; Seybold et al., 2015). In G1, the bridge’s distal end assembles the daughter SPB precursor, the satellite, which upon entry into a new cycle expands to form the duplication plaque and becomes embedded in the nuclear envelope. SPB inner plaque components are incorporated after insertion of the duplication plaque into the nuclear envelope (Adams and Kilmartin, 1999; Ruthnick and Schiebel, 2016). Although this duplication model suggests a conservative duplication process where a new SPB is assembled next to the old one, evidence also suggests that SPB is a dynamical structure that can grow and exchange. New subunits can be incorporated into SPB to increase its size, and old subunits can be replaced by new ones (Yoder et al., 2003; Greenland et al., 2010). The size of the SPB increases with the DNA content (Byers and Goetsch, 1975; Elliott et al., 1999; Chen et al., 2020). Moreover, the SPB responds differently to various cell cycle arrests, shrinking during α-factor-mediated G1 arrest and growing during a variety of G2 and M-phase arrests (Yoder et al., 2003; Jaspersen and Winey, 2004). While the mechanisms that limit SPB duplication once per cell cycle are extensively studied, the mechanisms that regulate SPB size and exchange remain mostly elusive.

Here, we identified Bud14 as a critical protein for maintaining SPB size. Using quantitative fluorescence microscopy, we show that cells lacking Bud14 have increased levels of inner, outer, and central plaque proteins at the SPBs during anaphase. Increased levels of inner and outer plaque proteins in *bud14Δ* cells were also observed during M-phase arrest mediated by the microtubule poison nocodazole. Intriguingly, during α-factor-dependent G1 arrest, inner and outer plaque proteins responded differently to the absence of *BUD14*. We further show that the interaction of Bud14 with Glc7, the Protein Phosphatase 1 (PP1), but not with the Kelch proteins, is indispensable for maintaining Spc110 levels at the SPBs. In addition, our data suggest that Bud14-Glc7 function limits SPB-bound levels of Spc110 at a point after entry into a new cell cycle. Thus, our work offers new insights into the mechanisms that regulate SPB size.

## 2. Materials and methods

### 2.1. Yeast strains, growth conditions, and cell cycle synchronizations

Yeast strains used in this study are listed in Table 1. All strains are isogenic to S288C. Basic yeast methods and growth media were as described by Sherman (1991). Chromosomal gene deletion and C-terminal tagging were performed using cassette PCR-based gene editing methods as described by Knop et al. (1999) and Janke et al. (2004). mCherry-*TUB1*-containing *URA3*-based yeast integration plasmid pAK011 was integrated into the genome at *ura3-52* locus (Kocakaplan et al., 2021). To obtain *bud14-F379A*, *bud14-F379A*-containing *LEU2-*based yeast integration plasmid (pSMG06) was integrated into the genome at *leu2Δ1* locus.

For α-factor-mediated G1 phase arrest, log-phase cultures were treated with 10 μg/mL α-factor (Sigma #T6901) for approximately 130 min. For synchronization in mitosis using nocodazole, α-factor-arrested cells were washed and released into α-factor-free, filter-sterilized YPAD media containing 15 μg/mL nocodazole (Sigma #M1404) and incubated for approximately 2 h. Cell cycle arrests were confirmed by microscopy after fixing the cells with 70% ethanol and resuspending them in PBS containing 1 μg/mL 4’,6-diamino-2-phenylindole (DAPI, Sigma).

### 2.2. Fluorescence microscopy

Fluorescence microscopy was performed using an Axio Observer 7 motorized inverted epifluorescence microscope (Carl ZEISS) with Axiocam 702 Monochrome camera, Colibri 7 LED light source, and filter sets 95 and 44 (Carl ZEISS). Images were acquired using 100× Plan Apochromat immersion oil objective and with 2×2 binning. For each view of field, 13 z-stacks of 0.30 μm thickness were acquired. In all experiments, LED intensity was 20% and the exposure time was 100 μs for sfGFP visualization.

Yeast cells were imaged live during the analysis of log-phase cultures, while cells involved in synchronization experiments were fixed with 8% PFA (Merck, 30525-89-4). For microscopy, all cells were grown in filter-sterilized SC-complete media, except for nocodazole arrest, which was performed in filter-sterilized YPAD media.

### 2.3. Fluorescence intensity quantifications and spindle length measurements

Image J (NIH, Bethesda, MD, USA) was used to analyze all microscopy images. For measuring the mean fluorescence intensities (FI) of sfGFP foci (region of interest, ROI), an area of 0.494 μm^2^ (24 pixels) was selected around the spindle poles, and FI was measured using ImageJ measure tool. As a background signal, the FI of an intracellular area free from sfGFP foci was measured. To obtain background-corrected FI, the background FI was subtracted from the FI of the ROI. Spindle length was measured using the ImageJ measure tool after drawing a line between the spindle poles.

### 2.4. Number of molecule calculations

Nuf2-sfGFP was used as a reference for calculating the number of molecules of SPB proteins C-terminally tagged with sfGFP (SPB-sfGFP). Log phase culture of the Nuf2-sfGFP was mixed with the log-phase culture of the sample of interest in 1/3 ratio before imaging to observe Nuf2-sfGFP and SPB-sfGFP in the same field of view. Samples containing SPB-sfGFP also contained mCherry-*TUB1* as a spindle marker, which allowed for the discrimination of cells with SPB-sfGFP from cells with Nuf2-sfGFP. FI of Nuf2-sfGFP and SPB-sfGFP were measured and corrected for background as explained above. Nuf2-sfGFP measurements were performed in cells in anaphase, based on pole-to-pole distances (pole-to-pole distance > 3μm). Median of background corrected FI Nuf2-sfGFP was considered as 352 molecules (Joglekar et al., 2006; Joglekar et al., 2008; Coffman et al., 2011; Lawrimore et al., 2011). The number of molecules of corresponding SPB-sfGFP at the SPBs was calculated by dividing the background-corrected FI of SPB-sfGFP to the median of background corrected FI of Nuf2-sfGFP, and then multiplying by 352.

Nuf2-sfGFP reference was also included in experiments where the results were presented as “relative fluorescence intensities”. Cells in these experiments were fixed before image analysis. Considering possible differential effects of fixation on Nuf2-sfGFP and SPB-sfGFP, we named the outcomes “relative fluorescence intensities” rather than number of molecules.

### 2.5. Protein methods

Preparation of total protein samples and immunoblotting were as previously described (Meitinger et al., 2016). Total cellular proteins were precipitated using trichloroacetic acid from cells grown to log-phase in YPAD. The primary antibodies utilized were rabbit anti-GFP (Abcam, ab290), mouse anti-HA (gift from Gislene Pereira), and rabbit anti-Tubulin (Abcam, EPR13799). The secondary antibodies used were goat anti-rabbit HRP-conjugated antibody (Advansta #R-05072-500) and goat anti-mouse HRP-conjugated antibody (Advansta #R-05071-500). Chemiluminescence signals were captured using the Bio-Rad Chemidoc MP system. To quantify total protein levels, protein bands were selected using the rectangular selection tool in ImageJ, and mean fluorescence intensities were quantified using the ImageJ measure tool. Same-sized areas were selected for the quantification of protein band intensities. In addition, a protein-free area above the bands was selected for background correction. Mean fluorescence intensities were corrected by the background signal by subtracting the background intensities from the protein band intensities. Corrected intensities of the SPB proteins were divided by the corrected intensities of Tubulin signals to calculate the relative total levels of steady-state proteins.

### 2.6. Statistical analysis and data presentation

GraphPad Prism 8.0.1 (GraphPad, Le Jolla, CA, USA) software was used for plotting graphs, obtaining descriptive statistics and performing statistical tests. ImageJ, Photoshop, and Illustrator 2024 (Adobe, San Jose, CA, USA) were used for brightness and contrast adjustment, as well as for the compilation and labeling of images.

## 3. Results

### 3.1. Lack of *BUD14* leads to elevated levels of SPB inner, central and outer plaque proteins at SPBs

Our previous work demonstrated an elevated presence of the signaling proteins Bfa1-Bub2 and Tem1 at the SPBs of yeast cells lacking Bud14 (Kocakaplan et al., 2021). As these proteins bind to the SPB structural proteins Nud1 and Spc72 (Gryaznova et al., 2016), we hypothesized that the increase in SPB-bound signaling proteins might be due to an augmented number of binding sites at the SPBs. Therefore, we sought to analyze SPB-bound levels of SPB structural proteins in wildtype cells (*BUD14*) and in cells lacking *BUD14* (*bud14Δ*). For this, we tagged a total of seven SPB structural protein belonging to the SPB inner plaque (Spc110, Spc97), central plaque (Scp42, Spc29), outer plaque (Spc72, Spc97 and Nud1), and the half-bridge (Sfi1) (Muller et al., 2005; Kilmartin, 2014) (Figure 1a) at their C-terminus with superfolding GFP (sfGFP) (Pédelacq et al., 2006). Gene tagging was performed at the endogenous loci. sfGFP fluorophore was chosen for its short maturation time. Cells also contained mCherry-*TUB1* as a spindle marker. We quantified the mean fluorescence intensities of indicated proteins at the SPBs of anaphase cells (spindle length ≥ 3 μm) that came from a log-phase culture. All proteins analyzed, except for the half-bridge component Sfi1, exhibited elevated mean fluorescence intensities in *bud14Δ* cells compared to the wildtype cells (Figures 1b–1h).

**Figure 2:**
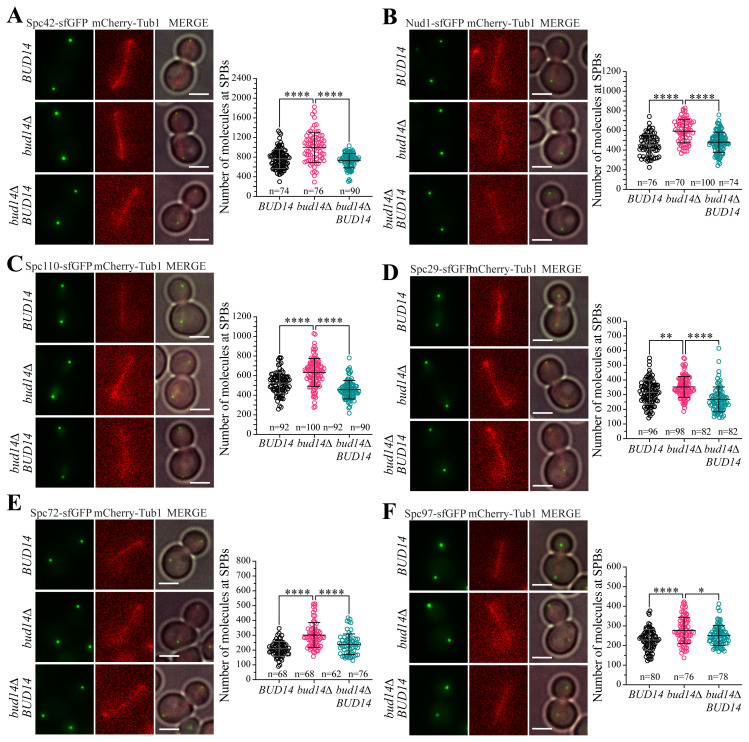
Number of molecules of SPB structural proteins at one SPB. **A–F**. Number of molecules of SPB-bound Spc42 (A), Nud1 (B), Spc110 (C), Spc29 (D), Spc72 (E), and Spc97 (F) in *BUD14* wildtype and *bud14Δ* cells, as well as *bud14Δ* cells complemented with wildtype *BUD14* (*bud14Δ BUD14*). Measurements come from cells at anaphase (spindle length ≥ 3μm). Representative microscopy images are shown. mCherry-Tub1 serves as the spindle marker. n: sample size. Scale bar: 3 μm. ****: p < 0.0001, **: p < 0.01, *: p < 0.05, according to one-way ANOVA.

In a different experimental setup, we quantified the number of SPB-bound molecules of SPB structural proteins using the kinetochore protein Nuf2, which has a known number of molecules, as a reference (Joglekar et al., 2006; Joglekar et al., 2008; Coffman et al., 2011; Lawrimore et al., 2011). Prior to the microscopy, we mixed log-phase cultures of Nuf2-sfGFP-containing cells and mCherry-*TUB1*-containing cells that had one of the SPB components tagged with sfGFP (SPB-sfGFP). mCherry-*TUB1* allowed us to distinguish SPB-sfGFP from Nuf2-sfGFP during fluorescence quantification. Mean fluorescence intensities of Nuf2-sfGFP and SPB-sfGFP were measured in the same acquired field. Given that 352 Nuf2 molecules form a kinetochore cluster near the spindle poles of yeast during anaphase (Coffman et al., 2011; Lawrimore et al., 2011), we employed a fluorescence ratio method to convert fluorescence intensities to number of molecules. The geometric mean of number of molecule values we obtained in wildtype cells were comparable with previously reported values for Spc97 and Spc72 (Erlemann et al., 2012), as well as with the suggested stoichiometry for Spc42:Spc29 (Muller et al., 2005; Viswanath et al., 2017) (Table 2, Figures 2a–2f). The geometric mean of Spc110 we obtained in wildtype cells were larger than the previously reported values (Erlemann et al., 2012), which may be due to differences in the contribution of the nucleoplasmic pool of Spc110 in the measurements. With this approach, too, we confirmed that Spc42, Nud1, Spc110, Spc29, Spc72, and Spc97 levels were increased at SPBs of *bud14Δ* cells in anaphase (Table 2, Figures 2a–2f). The steady-state total levels of these proteins, however, were not increased in *bud14Δ* cells (Figure S1), suggesting that elevated SPB-bound levels do not stem from increased expression of these proteins. Furthermore, complementation of *bud14Δ* cells by wildtype *BUD14* (*bud14Δ BUD14*) rescued the *bud14Δ* phenotype (Figures 2a–2f), supporting that the increased SPB-bound levels of SPB structural proteins results from the lack of *BUD14*.

### 3.2. α-factor treatment affects *bud14Δ* phenotype at the inner and outer plaques differentially

**Figure 3:**
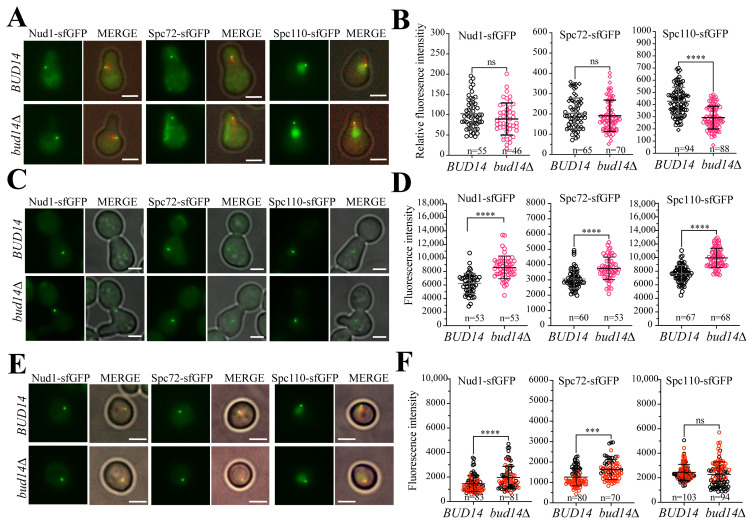
SPB-bound levels of SPB proteins in *bud14Δ* cells during different cell cycle stages. **A.** Representative images of Nud1-sfGFP, Spc72-sfGFP, and Spc110-sfGFP bearing wildtype and *bud14Δ* cells arrested in G1 using α-factor **B.** Fluorescence intensities of SPB-bound sfGFP-tagged proteins shown in A. **C.** Representative images of indicated wildtype and *bud14Δ* cells arrested in mitosis using the microtubule depolymerizing drug nocodazole. **D.** Relative fluorescence intensities of SPB-bound sfGFP-tagged proteins shown in C. **E.** Representative images of unbudded wildtype and *bud14Δ* cells that come from a log-phase culture. **F.** Relative fluorescence intensities of SPB-bound sfGFP-tagged proteins shown in E. Scale bar: 3 μm. ****: p < 0.0001 according to two-tailed Student’s *t*-test. n.s.: nonsignificant, p > 0.05.

The diameter of the SPB changes during the cell cycle (Bullitt et al., 1997). Accordingly, SPB grows during G2 and M-phase arrests and shrinks during α-factor-induced G1 arrest (Yoder et al., 2003; Jaspersen and Winey, 2004). We analyzed the inner and outer plaque proteins (Nud1, Spc72 and Spc110) in α-factor-arrested wildtype (*BUD14*) and *bud14Δ* populations. Intriguingly, α-factor-mediated G1 arrest resulted in equalization of Spc72 and Nud1 levels at the SPBs of wildtype and *bud14Δ* cells (Figures 3a and 3b). On the other hand, α-factor arrest caused a dramatic reduction in SPB-bound Spc110 levels in *bud14Δ* cells, such that less Spc110 was detected on the SPBs compared to wildtype cells (Figures 3a and 3b). We next synchronously released α-factor-arrested cells into nocodazole-containing fresh medium to obtain an M-phase arrest. During nocodazole-induced arrest, *bud14Δ* cells had more Nud1, Spc72, and Spc110 at their SPBs than the wildtype cells (Figures 3c and 3d). These data are in line with our previous conclusion that during M-phase, *bud14Δ* cells have more inner and outer SPB proteins localized at the SPBs. Furthermore, it suggests that during the α-factor-induced G1-arrest, inner and outer plaque proteins respond differently to the absence of *BUD14*. Equalization of Nud1 and Spc72 levels in wildtype and *bud14Δ* likely stems from shrinkage of SPB upon α-factor treatment rather than an effect of G1-phase because when we analyzed G1 cells (unbudded cells) from log-phase cultures, we observed that more Nud1 and Spc72 were present at the SPBs of *bud14Δ* cells compared to wildtype cells (Figures 3e and 3f). However, the effect observed in Spc110 during α-factor induced G1-arrest, is likely not solely due to the α-factor, because G1 cells (unbudded cells) from log-phase cultures had the same amount of Spc110 at the SPB in the absence and presence of *BUD14* (Figures 3e and 3f). We thus conclude that Bud14 exerts its effect on Spc110 at a point after entry into a new cell cycle and hereafter we focus on Spc110 regulation by Bud14.

### 3.3. Lack of Bud14-Glc7 interaction, but not the Kelch complex, causes altered levels of Spc110 at SPBs

**Figure 4:**
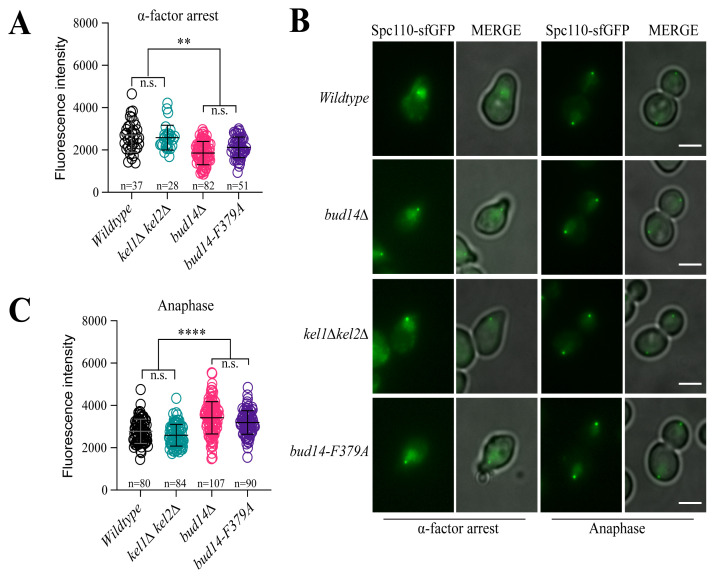
Effect of loss of Kel1-Kel2-Bud14 and Glc7-Bud14 on levels of Spc110 at SPBs. **A.** Fluorescence intensities of SPB-bound Spc110-sfGFP in wildtype, *kel1Δkel2Δ* and *bud14Δ* cell, as wells as cells the *bud14-F379A* mutant cells arrested in G1 with α-factor treatment. **B.** Representative still images. Scale bar: 3 μm. **C.** Fluorescence intensities of Spc110-sfGFP at SPBs of indicated cell types during anaphase (spindle length ≥ 3μm). n: sample size. ****: p < 0.0001, **: p < 0.01, according to one-way ANOVA. n.s.: nonsignificant, p > 0.05.

Bud14 forms a complex with two conserved Kelch proteins, Kel1 and Kel2 (Gould et al., 2014). Bud14-Kel1-Kel2 complex regulates the formin Bnr1 to control actin cable formation, polarized cell growth, and cytokinesis (Chesarone et al., 2009; Eskin et al., 2016). We investigated whether the role of Bud14 in SPB size maintenance is through its role in Bud14-Kel1-Kel2 complex. To address this, we measured SPB-bound Spc110-sfGFP levels in wildtype cells, in the *bud14*Δ mutant and in cells where *KEL1* and *KEL2* were deleted (*kel1Δ kel2Δ*) (Figures 4a–4c). Measurements were performed during α-factor-mediated G1 arrest (Figures 4a and 4b) and in anaphase cells from log-phase cell cultures (Figures 4b and 4c). We reasoned that if Bud14-Kel1-Kel2 complex were crucial regulating SPB-bound Spc110 levels by Bud14, then *kel1Δ kel2Δ* would phenocopy *bud14*Δ. However, unlike deletion of *BUD14*, deletion of *KEL1* and *KEL2* did not change SPB-bound levels of Spc110 in the analyzed conditions (Figures 4a–4c). We thus conclude that Bud14 is involved in maintenance of Spc110 levels at the SPBs independently from its function in the Bud14-Kel1-Kel2 complex.

Bud14 is a regulatory subunit of Glc7, sole member of the Protein Phosphatase 1 (PP1) family in budding yeast (Cullen and Sprague, 2002; Knaus et al., 2005; Lenssen et al., 2005). Therefore, we asked whether Bud14 exerts its effect on Spc110 via its role in Glc7 regulation. To address this question, we analyzed *bud14-F379A* mutant which cannot interact with Glc7 (Knaus et al., 2005; Kocakaplan et al., 2021). *bud14-F379A* mutant phenocopied *bud14Δ*, supporting that lack of Bud14-Glc7 interaction causes altered levels of Spc110 at SPBs (Figures 4a and 4c). Taken together, these data show that the role of Bud14 in regulation of Spc110 levels stems from its interaction with Glc7 but not with the Kelch proteins.

### 3.4. Increased Spc110 recruitment to the SPBs in the absence of Bud14-Glc7 coincides with early stages of the cell cycle

**Figure 5:**
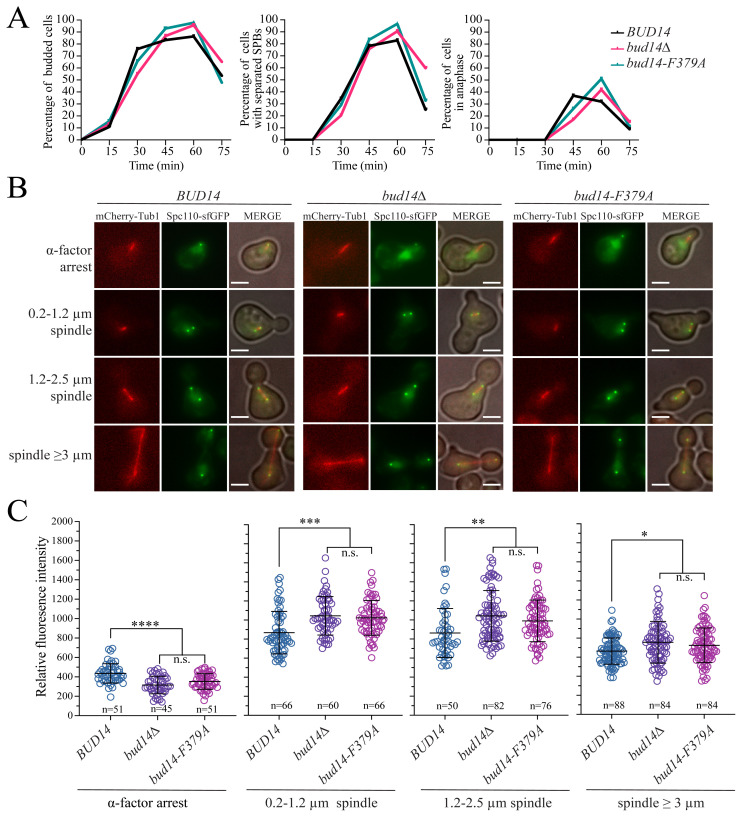
Analysis of cell-cycle-dependent changes in SPB-bound Spc110 levels. **A.** *SPC110-sfGFP mCherry-TUB1* bearing wildtype (*BUD14*), *bud14Δ* and *bud14-F379A* cells were synchronized in G1 using α-factor (t = 0) and released from G1 to allow synchronous cell cycle progression. Samples were collected every 15 min. Graphs showing the percentage of budded cells (indicating cells that passed G1/S), percentage of cells with separated SPBs (indicating cells that duplicated their SPBs based on Spc110-sfGFP), and percentage of cells in anaphase (spindle length ≥ 3 μm, based on mCherry-Tub1) were plotted. A minimum of 100 cells were counted at each time point. **B.** Representative still images of cells during different cell cycle stages. Spindle length was measured using mCherry-Tub1 as a spindle marker. Cells from timepoint zero (α-factor arrest), cells from timepoint 30 min with spindle length in the range of 0.2–1.2 μm, cells from time points 30 and 45 min with spindle length in the range of 1.3–2.5 μm, and cells from time points 45 and 60 min with spindle length ≥ 3 μm were categorized in indicated groups. Scale bar: 3 μm. **C.** Relative fluorescence intensities of SPB-bound Spc110-sfGFP. n: sample size. ****: p < 0.0001, ***: p < 0.001, **: p < 0.01, *: p < 0.05 according to one-way ANOVA. n.s.: nonsignificant, p > 0.05.

To understand when Spc110 levels increase at SPBs in the absence of Bud14-Glc7, we performed a time-course assay where wildtype, *bud14Δ*, and *bud14-F379A* cells, each containing *SPC110-sfGFP* and *mCherry-TUB1* were arrested in G1 by α-factor and then released from this arrest to allow synchronous cell cycle progression. Samples were collected every 15 min for about one cell cycle and analyzed by fluorescence microscopy. Based on budding, SPB separation and spindle elongation *bud14Δ* and *bud14-F379A* cells exhibited a slight delay in entry into cell cycle (budding and SPB separation) and anaphase onset (spindle elongation) after their release from the G1 arrest (Figure 5a).

In line with our previous result, less Spc110 were detected at the SPBs in *bud14*Δ and *bud14-F379A* cells compared to the wildtype cells, during α-factor-mediated G1 arrest (Figures 5b and 5c). In all cell types analyzed, Spc110 levels increased after release from the G1 arrest. However, in *bud14*Δ and *bud14-F379A* cells, Spc110 levels increased more than in wildtype cells and exceeded wildtype levels after SPB duplication (defined by small-budded cells with a spindle length of 0.2–1.2 μm) (Figures 5b and 5c). Upon entry into anaphase (spindle length > 3 μm), Spc110 levels dropped in wildtype cells (Figures 5b and 5c). This cell-cycle-dependent increase and decrease in the SPB-bound Spc110 is in concordance with previous reports (Yoder et al., 2003; Erlemann et al., 2012). With the anaphase onset (spindle length > 3 μm), levels of Spc110 also decreased at SPBs of *bud14*Δ and *bud14-F379A* cells; however, it remained higher than in the wildtype cells (Figures 5b and 5c). This result suggests that lack of Bud14-Glc7 leads to an increase in the SPB-bound levels of Spc110 starting from the early stages of the cell cycle, which may be concurrent with Spc110 loading on the SPB during SPB duplication.

## 4. Discussion

SPBs, the functional equivalent of centrosomes in the yeast, have been a great model to understand centrosome function and acentriolar centrosome biogenesis. A new SPB is assembled next to the old one in every cell cycle, suggesting a conservative duplication model, yet SPBs are dynamic and thus can grow and exchange. Although mechanisms that limit SPB duplication to once per cell cycle are well studied in *S. cerevisiae*, very little is known about mechanisms that regulate the size of SPB. Here, we identified Bud14 as a critical protein that play a role in limiting the SPB size of the budding yeast.

Based on quantitative fluorescence microscopy data, we observed more Spc110, Spc97, Scp42, Spc29, Spc72, and Nud1 at the SPBs of *bud14Δ* cells compared to wildtype cells. SPB-bound levels of the half-bridge component Sfi1, however, did not significantly change upon *BUD14* deletion. Although the low florescence signal of Sfi1-sfGFP at the SPBs may preclude detection of small differences, these data altogether suggest the presence of a larger outer, inner, and central plaque in *bud14Δ* cells, whereas the size of the half-bridge is likely not affected. Notably, fold increase in number of inner, outer, and central plaque proteins at SPBs upon *BUD14* deletion varied among analyzed proteins, ranging from 1,1- to 1,3-fold. These differences may indicate that different layers of the SPBs may be differentially affected by the absence of Bud14. Alternatively, they may stem from possible differences in fluorescence quenching of fluorophores depending on how they are packed and oriented, whereas the fold increase of SPB layers may remain the same. Electron microscopy-based analysis of layers’ thicknesses and lateral lengths will be necessary to understand how and to what extent SPB size is changed in the absence of Bud14.

What are the mechanisms by which Bud14 impact on SPB size? We think that Bud14 may have an impact on loading and/or organization of SPB inner, central, and outer plaque proteins rather than the duplication process of SPB. SPB is built around the Spc42 core which organizes into a hexagonal array (Drennan et al., 2019). Spc42 overexpression results in lateral expansion of the central plaque (Bullitt et al., 1997), cooverexpression of Spc42, Spc29, and Spc110 increases the size of the inner plaque (Elliott et al., 1999). Of importance, *BUD14* deletion did not cause an increase in steady-state protein levels of the SPB structural proteins, which rules out the possibility of a regulation at the level of protein expression. Given our data that Bud14 works with the PP1 (Glc7) in regulation of Spc110 levels at the SPBs, we favor that the role of Bud14 in regulation of SPB size is via its interaction with Glc7. It is tempting to speculate that Bud14-Glc7 may dephosphorylate one or more SPB proteins, or key proteins that regulate SPBs, to restrict the size of the SPB in every cell cycle. Indeed, most of the SPB structural proteins are heavily phosphorylated (Geymonat et al., 2020; Lanz et al., 2021; Zhou et al., 2021; Abbasi et al., 2022) and more is known on kinases than phosphatases that act on these proteins. Additionally, data from existing literature indicate that it is plausible for Glc7-Bud14 to regulate SPB-associated proteins: We have previously showed that Bud14-Glc7 interacts with and dephosphorylates Bfa1, a cell cycle checkpoint protein that uses SPBs as a scaffold (Kocakaplan et al., 2021). Other studies have identified Bud14 in close proximity to SPB-associated proteins, namely Mob1 and Dbf2 (Hruby et al., 2011; Zhou et al., 2021). Furthermore, although Bud14 is not yet detected to be enriched at SPBs through classical direct fluorescence microscopy methods (our unpublished data), Glc7 is enriched around the spindle poles (Bloecher and Tatchell, 2000) indicating possible interactions therein. Nevertheless, more work needs to be done to understand whether Bud14 directly interacts with the core SPB proteins and, if so, where this interaction takes place.

Centrosomes do not only serve as microtubule organizing centers but also function as scaffolds for many signaling pathways (Arquint et al., 2014; Chan et al., 2017; Langlois-Lemay and D’Amours, 2022; Lin et al., 2022). In budding yeast, both the Mitotic Exit Network (MEN) and for the Spindle Position Checkpoint (SPOC) proteins dock onto SPBs through direct interaction with the SPB outer plaque proteins (Gruneberg et al., 2000; Rock et al., 2013; Gryaznova et al., 2016). Binding of MEN proteins to the SPBs is critical for mitotic exit to take place, whereas depletion of key MEN proteins from SPBs is crucial for the anaphase arrest imposed by the SPOC. Indeed Bud14-Glc7 dephosphorylation of Bfa1 has been reported to be essential for the functioning of SPOC (Kocakaplan et al., 2021). Accordingly, cells with impaired Bud14-Glc7 fail to arrest in anaphase upon spindle mispositioning. Thus, limitation of the SPB outer plaque size by Bud14 may be an additional mechanism by which Bud14 impinges on the SPOC.

## Supplementary Data

Figure S1 Effect of Bud14 on steady-state levels of SPB structural proteins **A.** Immunoblot showing levels of Spc42-sfGFP, Spc72-sfGFP, Spc29-sfGFP and Spc97-sfGFP in WT (+) and in *bud14Δ* cells (−). **B.** Immunoblot showing levels of Nud1-3HA and Spc110-3HA in WT (+) and in *bud14Δ* cells. Tubulin served as loading control. Note that GFP antibody recognized an unspecific band around Nud1-sfGFP and Spc110-sfGFP, and thus levels of these proteins were shown using the HA epitope and and-HA antibody. **C.** Ratio of SPB structural protein band intensities to the Tubulin band intensities. Graph shows mean of three experiments. Error bars are standard deviation. p>0.05 according to two-tailed student’s t-test.

## Figures and Tables

**Table 1:** Table of yeast strains used in this study.

Strain name	Description	Reference
ESM356	*MATa ura3-52 leu2Δ1 his3Δ200 trp1Δ63*	(Pereira and Schiebel, 2001)
SGY024-1	ESM356 *SPC42-sfGFP-kanMX6 ura3-52::URA3-mCherry-TUB1*	This study
SGY025-1	ESM356 *SPC72-sfGFP-kanMX6 ura3-52::URA3-mCherry-TUB1*	This study
SGY026-1	ESM356 *SPC110-sfGFP-kanMX6 ura3-52::URA3-mCherry-TUB1*	This study
SGY027-1	ESM356 *NUD1-sfGFP-kanMX6 ura3-52::URA3-mCherry-TUB1*	This study
SGY031-1	ESM356 *SPC29-sfGFP-kanMX6 ura3-52::URA3-mCherry-TUB1*	This study
SGY032-1	ESM356 *SPC97-sfGFP-kanMX6 ura3-52::URA3-mCherry-TUB1*	This study
SGY037-1	ESM356 *SPC110-sfGFP-kanMX6 ura3-52::URA3-mCherry-TUB1 bud14Δ::klTRP1*	This study
SGY034-1	ESM356 *NUD1-sfGFP-kanMX6 ura3-52::URA3-mCherry-TUB1 bud14Δ::klTRP1*	This study
SGY038-1	ESM356 *SPC29-sfGFP-kanMX6 ura3-52::URA3-mCherry-TUB1 bud14Δ::klTRP1*	This study
SGY039-1	ESM356 *SPC97-sfGFP-kanMX6 ura3-52::URA3-mCherry-TUB1 bud14Δ::klTRP1*	This study
SGY046-1	ESM356 *SFI1-sfGFP-kanMX6 ura3-52::URA3-mCherry-TUB1*	This study
SGY048-1	ESM356 *SPC42-sfGFP-kanMX6 ura3-52::URA3-mCherry-TUB1 bud14Δ::klTRP1*	This study
SGY050-1	ESM356 *SPC72-sfGFP-kanMX6 ura3-52::URA3-mCherry-TUB1 bud14Δ::klTRP1*	This study
SGY052-1	ESM356 *NUF2-sfGFP-kanMX6*	This study
SGY058-1	ESM356 *bud14Δ::klTRP1 ura3-52::URA3-mCherry-TUB1 SFI1-sfGFP-kanMX6*	This study
SGY113-1	ESM356 *bud14Δ::klTRP1 ura3-52::URA3-mCherry-TUB1 leu2Δ1::LEU2-BUD14 SPC110-sfGFP-kanMX6*	This study
SGY114-1	ESM356 *bud14Δ::klTRP1 ura3-52::URA3-mCherry-TUB1 leu2Δ1::LEU2-bud14-F379A SPC110-sfGFP-kanMX6*	This study
SGY134	ESM356 *kel1Δ::his3MX6 kel2Δ::hphNT1 SPC110-sfGFP-kanMX6*	This study
AKY4042	*ESM356 NUD1-6HA-klTRP1*	This study
AKY4043	*ESM356 bud14Δ::his3MX6 NUD1-6HA-klTRP1*	This study
SGY150	*ESM356 SPC110-6HA-hphNT1*	This study
SGY151	*ESM356 bud14Δ::klTRP1 SPC110-6HA-hphNT1*	This study

**Table 2:** Number of Molecules of SPB structural proteins at the SPB.

Protein	Geometric mean of number of molecules at one SPB in anaphase ± Standard deviation	Fold increase in *bud14Δ*
**Spc42**	756.8 ± 1.3	1.3
**Nud1**	463.4 ± 1.2	1.3
**Spc110**	530.1 ± 1.2	1.2
**Spc29**	319.2 ± 1.3	1.1
**Spc72**	211.4 ± 1,3	1.3
**Spc97**	232.3 ± 1.2	1.2
