# Supplementary figures and images for: Bud14 function is crucial for spindle pole body size maintenance
Source: Turk J Biol. 2024 Aug 5;48(4):267–78. doi: 10.55730/1300-0152.2702 (PMC11407341; doi:10.55730/1300-0152.2702)

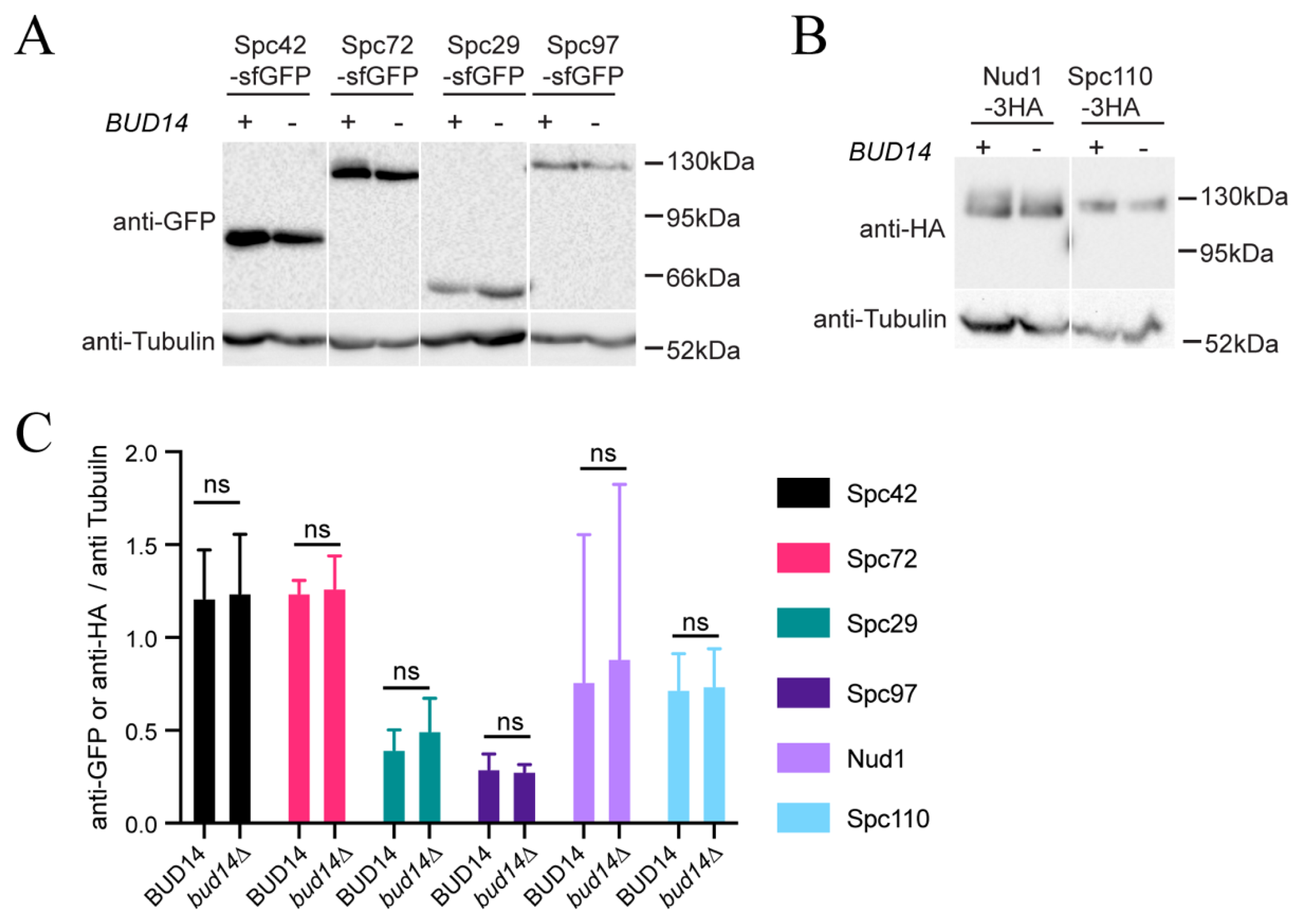

Supplement: Figure S1 — A. Immunoblot showing levels of Spc42-sfGFP, Spc72-sfGFP, Spc29-sfGFP and Spc97-sfGFP in WT (+) and in bud14Δ cells (−). B. Immunoblot showing levels of Nud1-3HA and Spc110-3HA in WT (+) and in bud14Δ cells. Tubulin served as loading control. Note that GFP antibody recognized an unspecific band around Nud1-sfGFP and Spc110-sfGFP, and thus levels of these proteins were shown using the HA epitope and and-HA antibody. C. Ratio of SPB structural protein band intensities to the Tubulin band intensities. Graph shows mean of three experiments. Error bars are standard deviation. p>0.05 according to two-tailed student’s t-test. [file tjb-48-04-267s1.tif]
